# Long‐term administration of pyridostigmine attenuates pressure overload‐induced cardiac hypertrophy by inhibiting calcineurin signalling

**DOI:** 10.1111/jcmm.13133

**Published:** 2017-03-10

**Authors:** Yi Lu, Ming Zhao, Jin‐Jun Liu, Xi He, Xiao‐Jiang Yu, Long‐Zhu Liu, Lei Sun, Li‐Na Chen, Wei‐Jin Zang

**Affiliations:** ^1^ Department of Pharmacology School of Basic Medical Sciences Xian Jiaotong University Health Science Center Xi'an Shaanxi China

**Keywords:** calcineurin, cardiac hypertrophy, pyridostigmine, acetylcholinesterase

## Abstract

Cardiac hypertrophy is associated with autonomic imbalance, characterized by enhanced sympathetic activity and withdrawal of parasympathetic control. Increased parasympathetic function improves ventricular performance. However, whether pyridostigmine, a reversible acetylcholinesterase inhibitor, can offset cardiac hypertrophy induced by pressure overload remains unclear. Hence, this study aimed to determine whether pyridostigmine can ameliorate pressure overload‐induced cardiac hypertrophy and identify the underlying mechanisms. Rats were subjected to either sham or constriction of abdominal aorta surgery and treated with or without pyridostigmine for 8 weeks. Vagal activity and cardiac function were determined using PowerLab. Cardiac hypertrophy was evaluated using various histological stains. Protein markers for cardiac hypertrophy were quantitated by Western blot and immunoprecipitation. Pressure overload resulted in a marked reduction in vagal discharge and a profound increase in cardiac hypertrophy index and cardiac dysfunction. Pyridostigmine increased the acetylcholine levels by inhibiting acetylcholinesterase in rats with pressure overload. Pyridostigmine significantly attenuated cardiac hypertrophy based on reduction in left ventricular weight/body weight, suppression of the levels of atrial natriuretic peptide, brain natriuretic peptide and β‐myosin heavy chain, and a reduction in cardiac fibrosis. These effects were accompanied by marked improvement of cardiac function. Additionally, pyridostigmine inhibited the CaN/NFAT3/GATA4 pathway and suppressed Orai1/STIM1 complex formation. In conclusion, pressure overload resulted in cardiac hypertrophy, cardiac dysfunction and a significant reduction in vagal discharge. Pyridostigmine attenuated cardiac hypertrophy and improved cardiac function, which was related to improved cholinergic transmission efficiency (decreased acetylcholinesterase and increased acetylcholine), inhibition of the CaN/NFAT3/GATA4 pathway and suppression of the interaction of Orai1/STIM1.

## Introduction

Cardiac hypertrophy remains a major cause of mortality in patients with cardiovascular diseases worldwide and has been recognized as a risk factor for heart failure characterized by sympathetic dominance and parasympathetic (vagal) withdrawal 1, 2, 3. Activation of the parasympathetic nervous system may have beneficial implications for treatment of cardiovascular disease 4. Previous clinical and experimental studies have shown that chronic vagal nerve stimulation enhances vagal tone and improves cardiac function 5, 6. Pyridostigmine, a reversible acetylcholinesterase (AChE) inhibitor, can prevent the hydrolysis of acetylcholine (ACh) and enhance the efficiency of cholinergic transmission. Several studies showed that pyridostigmine could increase heart rate variability and baroreflex sensitivity in normal rats and inhibit cardiac dysfunction during mental stress in healthy individuals 7, 8. Cholinergic stimulation with pyridostigmine reduces ventricular arrhythmia and repolarization in individuals with coronal artery disease and improves cardiac performance 9. Work in our laboratory has revealed that treatment with pyridostigmine ameliorates cardiac fibrosis and improves cardiac function in rats after myocardial infarction 10. However, whether pyridostigmine can protect against cardiac hypertrophy induced by pressure overload remains unclear.

Multiple intracellular signal transduction pathways, which are coupled with transcription factors in the nucleus for regulation of gene expression, are associated with cardiac hypertrophy 11. Molkentin *et al*. showed that the calcineurin (CaN)/nuclear factor of activated T cells 3 (NFAT3) pathway plays a critical role in cardiac hypertrophy 12. Activation of CaN leads to dephosphorylation and translocation of NFAT3 from the cytoplasm to the nucleus, which potentiates GATA4 and ultimately leads to transcription of genes encoding factors involved in hypertrophy 13. Several lines of evidence suggest that inhibition of the CaN/NFAT3/GATA4 pathway attenuates cardiac hypertrophy in rats after myocardial infarction 14, 15. A recent study suggested that stromal interaction molecule 1 (STIM1) and Orai1 are store‐operated channel molecules that play important roles in regulating the hypertrophic growth of cardiomyocytes 16. STIM1, which is located in the sarcoplasmic reticulum/endoplasmic reticulum and functions as a calcium‐(Ca^2+^) sensor, is activated when in close proximity to Orai1 in the plasma membrane and triggers Ca^2+^ influx 17. Interestingly, STIM1 and Orai1 mediate histamine‐evoked CaN/NFAT signalling, and decreased expression of STIM1 or Orai1 interrupts activation of the CaN/NFAT pathway in human umbilical vein endothelial cells 18. The development of novel pharmacological therapeutics that can prevent Oria1/STIM1 puncta formation and inhibit the CaN/NFAT3 pathway may afford better protection against cardiac hypertrophy and heart failure.

Therefore, this study examined changes in vagal discharge and investigated whether pyridostigmine can inhibit CaN/NFAT3/GATA4 and suppress Orai1/STIM1 complex formation as well as improve cardiac function in pressure overload‐induced cardiac hypertrophy.

## Materials and methods

### Animals

Adult male Sprague Dawley rats (8–10 weeks old), supplied by the Experimental Animal Centre of Xi'an Jiaotong University, were used in this study. All experimental procedures were performed in accordance with the Guide for the Care and Use of Laboratory Animals published by the National Institutes of Health (NIH publication No. 85‐23, revised 1996) and approved by the Ethics Committee of Xi'an Jiaotong University.

### Abdominal aorta constriction and telemetry transmitter implantation

Cardiac hypertrophy was induced by abdominal aorta constriction (AAC) as described previously 19. Briefly, rats were fasted for 12 hrs before anaesthesia (pentobarbital sodium, 45 mg/kg, i.p.). The abdomen and right vagal nerve neck area were shaved and then sprayed and wiped with a disinfectant antibacterial solution. The aorta was then dissected above the two renal arteries, and a silver clip (0.70 mm internal diameter) was placed on the exposed aorta above the level of the left renal artery.

The transmitter was placed within the abdominal cavity and tied to the abdominal muscle wall using a suture. The right cervical vagus nerve was identified, and the bipolar coiled electrode leads connected to the transmitter for recording vagal discharge were tunnelled underneath the skin and attached to the vagus nerve. Next, one electrode lead for the electrocardiograph recording was fixed to the xiphoid process and the other electrode was subcutaneously tunnelled through to the upper insertion of the sternohyoid muscle. The incision was then closed with sutures, and the animals were allowed to recover for 1 week.

**Figure 1:**
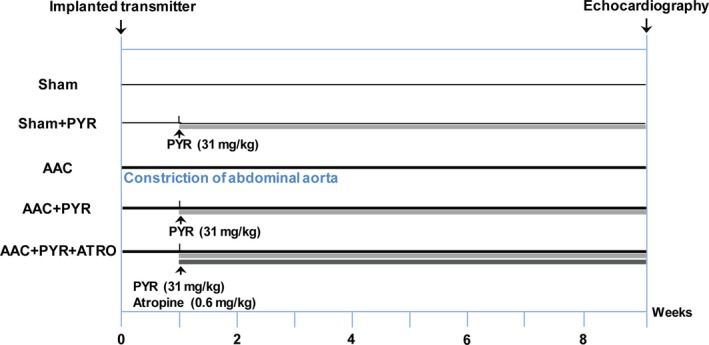
Experimental strategy and schedule. Time course of the experimental procedures. Sham, abdominal aorta constriction without clip placement; sham+PYR, sham with pyridostigmine; AAC, abdominal aorta constriction; AAC+PYR, AAC with pyridostigmine treatment; AAC+PYR+ATRO, AAC with pyridostigmine administration and intraperitoneal injection of atropine.

All rats were randomly divided into five groups of seven rats each after the surgical procedure: the AAC group, abdominal aorta constriction with pyridostigmine bromide treatment (AAC+PYR) group, abdominal aorta constriction with pyridostigmine and atropine (AAC+PYR+ATRO) group, sham group and sham with pyridostigmine (sham+PYR) group, which was subjected to the same surgical procedure without clip placement. The AAC+PYR and sham+PYR rats were administered pyridostigmine (31 mg/kg/day; Shanghai Zhongxi Sunve Pharmaceutical Co. Ltd., Shanghai, China) once per day for 8 weeks, and the AAC+PYR+ATRO rats received atropine (0.6 mg/kg/day; Sigma‐Aldrich, St Louis, MO, USA) by intraperitoneal injection before pyridostigmine (31 mg/kg/day) once per day for 8 weeks (Fig. 1).

### Electrophysiological recordings

After 1 week of recovery, the telemetry transmitter was switched on and vagal discharge and an electrocardiograph were recorded using the PowerLab data acquisition system (PowerLab/4SP; AD Instruments, Sydney, NSW, Australia) at least three times per week for 8 weeks.

### Echocardiography

Transthoracic echocardiograms to determine cardiac volume and left ventricular (LV) contractility were performed (Philips Medical Systems, Netherlands) after 8 weeks. In brief, rats were lightly anesthetized with pentobarbital sodium (30 mg/kg, i.p.) and placed on a heating pad for transthoracic echocardiography. The LV ejection fraction (EF), LV fractional shortening (FS), heart rate (HR), systemic vascular resistance (SVR), LV internal dimension in systole and diastole (LVIDs and LVIDd, respectively), end‐systolic and end‐diastolic LV volume (LVEDV and LVESV, respectively), thickness of the interventricular septum in systole and diastole (IVSs and IVSd, respectively) and thickness of the LV posterior wall in systole and diastole (LVPWs and LVPWd, respectively) were obtained from the echocardiograms.

### Haemodynamic parameters

Haemodynamic parameters were recorded and evaluated using PowerLab (AD Instruments) after AAC for 8 weeks. All rats were anaesthetized with pentobarbital sodium (45 mg/kg, i.p.) until the eyelid reflex was lost. The right carotid artery was isolated and pressure transducers were introduced into the left ventricle to measure heart rate (HR), LV systolic pressure (LVSP), LV end‐diastolic pressure (LVEDP) and maximum slope of systolic pressure increment and diastolic pressure decrement (+dp/dt and −dp/dt, respectively). A heating pad was used to maintain body temperature during this process. All haemodynamic parameters were considered significant after a 30‐min. steady‐state condition.

### Preparation of blood and tissue samples

At the end of the experiments, all rats were killed by exsanguination under anaesthesia with pentobarbital sodium 20. Blood drawn from the abdominal aorta was collected and centrifuged at 5000 × *g* for 10 min., and the serum was aspirated for further analysis.

All hearts were excised rapidly and washed with 4°C phosphate‐buffered saline (137 mM NaCl, 2.7 mM KCl, 10 mM Na_2_HPO_4_, and 2 mM KH_2_PO_4_ at pH 7.4). The right ventricular free wall and atrial appendages were dissected away before weighing the left ventricle. The remaining left ventricles were snap‐frozen in liquid nitrogen and stored at −80°C.

### Histological and morphological analyses of cardiac hypertrophy

All hearts were removed, washed, photographed and cut into three transverse sections parallel to the atrioventricular groove. Transverse sections of left ventricles were fixed in 4% formalin, embedded in paraffin, cut into 5‐μm sections and stained with haematoxylin and eosin (HE) and Masson's trichrome for histological examination. The myocyte cross‐sectional diameter and fibrosis area were measured using Image‐Pro Plus 6.0 (Media Cybernetics, Silver Spring, MD, USA). The diameters of cardiomyocytes were evaluated in 20 visual fields selected randomly. Fibrosis area percentages were determined as the ratios between interstitial collagen deposition in the sham group and in the other groups.

### Measurement of serum ACh and AChE levels

Serum concentrations of ACh and AChE activity were determined using a commercially available kit (Jiancheng Bioengineering Institute, Nanjing, China). According to the manufacturer's instructions, the serum was mixed with kit reagents, and the absorbance at 550 and 412 nm of the samples was determined using a microplate spectrophotometer (Beyotime Institute of Biotechnology, Jiangsu, China).

### Western blot

Protein was extracted from approximately 50 mg of myocardial tissue, and the concentration in each sample was determined using a BCA protein assay kit (Beyotime). Protein samples (30 μg) were separated by 10% SDS‐PAGE and transferred to a PVDF membrane (Millipore, Billerica, MA, USA). The membranes were incubated in a blocking solution of Tris‐buffered saline containing 0.1% Tween (TBST) containing 5% non‐fat dry milk for 1 hr at room temperature and incubated with the following primary antibodies: rabbit polyclonal to Orai1 (Santa Cruz Biotechnology, Santa Cruz, CA, USA), rabbit monoclonal to STIM1 (Cell Signaling Technology, Inc., St. Louis Park, MN, USA), rabbit monoclonal to CaN (GeneTex, Inc., San Antonio, TX, USA), rabbit polyclonal to NFAT3 (Abcam, Cambridge, UK), rabbit polyclonal to GATA4 (phosphor S105; Abcam), rabbit polyclonal to GATA4 (Bioworld Technology, Co., Ltd, Nanjing, China), goat polyclonal to β‐myosin heavy chain (β‐MHC; Santa Cruz Biotechnology), rabbit polyclonal to atrial natriuretic peptide (ANP; Santa Cruz Biotechnology), goat polyclonal to brain natriuretic peptide (BNP; Santa Cruz Biotechnology) and mouse monoclonal to GAPDH (Cell Signal Pathway Research Tools Supplier, CMCTAG, Inc.,Milwaukee, WI, USA). After being washed six times with TBST, the membranes were incubated for 35 min. with a peroxidase‐conjugated rabbit anti‐goat IgG (Signalway Antibody LLC, College Park, MD, USA), rabbit anti‐mouse IgG (Signalway Antibody LLC) and goat anti‐rabbit IgG (ZSGB‐BIO, Beijing, China). Chemiluminescence was detected using an ECL‐Plus kit (Millipore) and exposure to X‐ray film. Bands were quantified by densitometry using the Quantity One software (Bio‐Rad Laboratories, Berkeley, CA, USA).

### Immunoprecipitation

Protein (500 μg) was incubated with Orai1 (Santa Cruz Biotechnology) and a rabbit monoclonal antibody to STIM1 (Cell Signaling Technology) overnight. The next day, the membrane was incubated with pre‐washed A/G agarose beds (50 μl) at 4°C for 3 hrs and washed four times with lysate buffer. Samples (30 μg) were analysed by Western blotting.

### Statistical analysis

Quantitative data are expressed as mean ± S.E.M. One‐way anova followed by Tukey's multiple comparison test was performed to determine the significance of differences; *P* < 0.05 was defined as statistically significant.

## Results

### Effect of pyridostigmine on vagal activity in rats with cardiac hypertrophy

**Figure 2:**
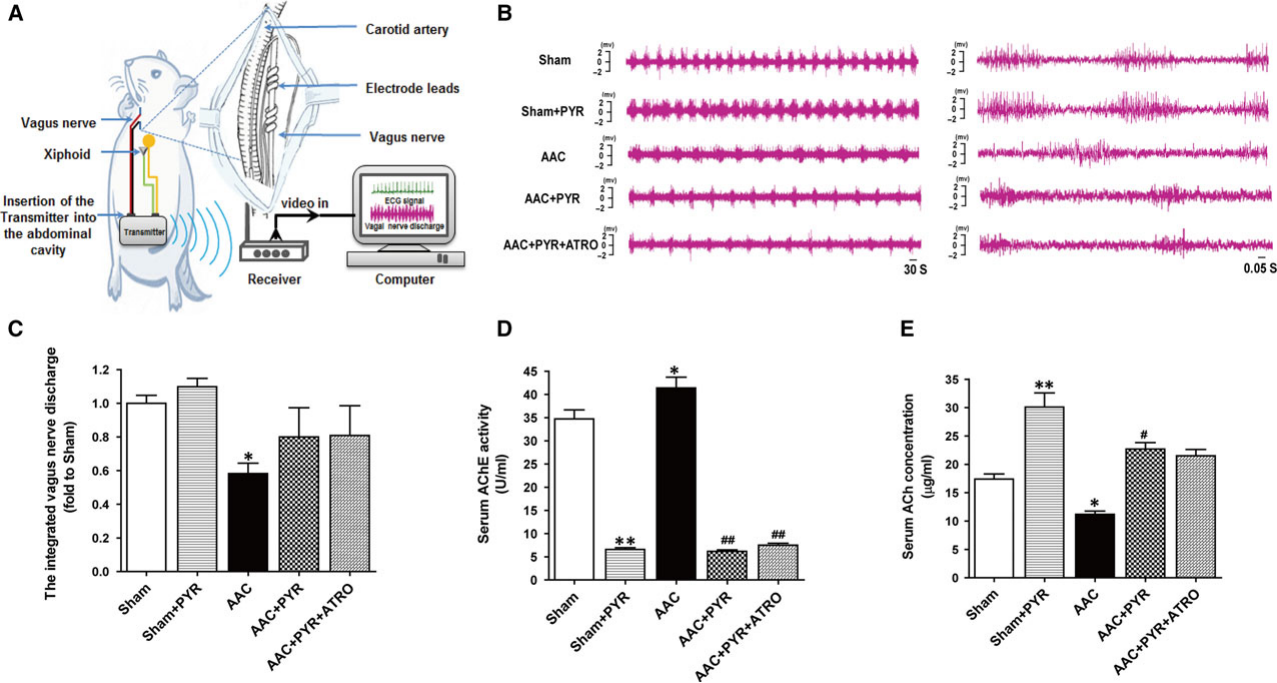
Effect of pyridostigmine on vagal activity in rats with cardiac hypertrophy. (**A**) Telemetry transmitter implantation and discharge recording. (**B**) Representative sample recordings of the discharge of the vagus nerve in rats. (**C**) The integrated vagus nerve discharge (*n* = 5). (**D** and **E**) Serum AChE and ACh levels. Data are means ± S.E.M. *P < 0.05, **P < 0.01 *versus* sham. #*P* < 0.05, ##*P* < 0.01 *versus* AAC.

Spontaneous discharges of the vagus nerve in rats were recorded (Fig. 2A). The discharge frequency of the vagus nerve was significantly lower in the AAC than sham group (Fig. 2B). There was no difference in discharge frequency between the AAC and AAC+PYR groups. Integrated vagal discharge in the AAC group was lower than that in the sham group (Fig. 2C). Treatment with pyridostigmine did not affect integrated vagal discharge.

The serum concentration of ACh in the sham+PYR group differed significantly from that in the sham group (Fig. 2E). It was significantly lower in the AAC than in the sham group; this reduction was reversed by pyridostigmine. In contrast, AChE activity was markedly lower in the sham+PYR group than in the sham group (Fig. 2D). Atropine did not attenuate the effect of pyridostigmine on serum AChE activity (Fig. S1). AChE activity was significantly higher in the AAC than in the sham group, but pyridostigmine administration reduced it in the AAC+PYR and AAC+PYR+ATRO groups.

### Pyridostigmine treatment improved cardiac function in rats with pressure overload

**Figure 3:**
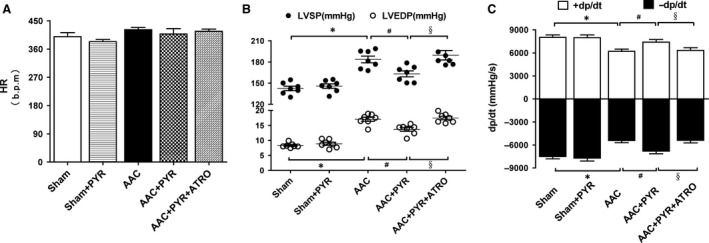
Pyridostigmine improved haemodynamic parameters in cardiac hypertrophy induced by chronic pressure overload. (**A**) HR, heart rate. (**B**) LVSP, left ventricular systolic pressure. LVEDP, left ventricular end‐diastolic pressure. (**C**) ±dp/dt, maximum slope of systolic pressure increment and diastolic pressure decrement. Data are means ± S.E.M. (*n* = 7). *P < 0.05 *versus* sham. #*P* < 0.05 *versus* AAC. §*P* < 0.05 *versus* AAC+PYR.

Haemodynamic parameters were recorded and analysed. No marked changes in HR were observed among the groups (Fig. 3A). LVSP and LVEDP significantly increased in the AAC group compared with the sham group; treatment with pyridostigmine reduced the dysfunction in rats with pressure overload (Fig. 3B). The ±dp/dt was significantly reduced in the AAC group, and reversed by treatment with pyridostigmine. (Fig. 3C). There were no significant differences in LVSP, LVEDP and ±dp/dt between the sham+PYR and sham groups (Fig. 3). Echocardiograms revealed no significant differences in EF, FS, HR or SVR among groups (Table 1 and Table S1).

**Table 1:** Echocardiography measurement in pyridostigmine‐treated rats with pressure overload

	Sham	Sham+PYR	AAC	AAC+PYR	AAC+PYR+ATRO
EF(%)	85.20 ± 2.61	85.34 ± 3.48	79.48 ± 4.55	83.15 ± 5.93	78.88 ± 6.40
FS(%)	49.09 ± 3.04	49.46 ± 4.34	43.06 ± 4.55	47.35 ± 6.88	42.94 ± 6.00
HR(bpm)	374.14 ± 21.81	370.86 ± 24.27	379.14 ± 20.23	373.57 ± 13.59	374.57 ± 16.08
SVR(dyne×sec/cm^5^)	146.01 ± 15.93	142.69 ± 10.93	159.03 ± 18.65	151.31 ± 13.57	156.26 ± 16.98
LVIDd(mm)	6.26 ± 0. 44	6.45 ± 0.16	7.32 ± 0.14*	6.49 ± 0.41#	7.19 ± 0.32§
LVIDs(mm)	3.18 ± 0.13	3.26 ± 0.28	4.16 ± 0.29*	3.42 ± 0.50#	4.09 ± 0.28§
LVEDV(ml)	0.57 ± 0.11	0.62 ± 0.04	0.88 ± 0.05*	0.63 ± 0.11#	0.84 ± 0.10§
LVESV(ml)	0.08 ± 0.01	0.09 ± 0.02	0.18 ± 0.04*	0.11 ± 0.04#	0.17 ± 0.03§
LVPWd(mm)	1.71 ± 0.06	1.72 ± 0.12	2.33 ± 0.40*	1.81 ± 0.23#	2.47 ± 0.14§
LVPWs(mm)	2.50 ± 0.30	2.59 ± 0.21	3.58 ± 0.28*	2.68 ± 0.30#	3.42 ± 0.35§
IVSd(mm)	1.84 ± 0.24	1.86 ± 0.17	2.47 ± 0.23*	1.97 ± 0.20#	2.50 ± 0.17§
IVSs(mm)	2.49 ± 0.36	2.54 ± 0.32	3.17 ± 0.32*	2.87 ± 0.20#	3.49 ± 0.18§

EF, left ventricular ejection fraction; FS, left ventricular fractional shortening; HR, heart rate; SVR, systemic vascular resistance; LVIDd(s), left ventricular internal dimension in systole and diastole; LVED(S)V, end‐systolic and end‐diastolic left ventricular volumes; IVSd(s), thickness of the interventricular septum; LVPWd(s), thickness of the left ventricular posterior wall in systole and diastole. Data are shown as the means ± standard error of the mean (*n* = 7). *P < 0.05 *versus* sham. ^#^ *P* < 0.05 *versus* AAC. ^§^ *P* < 0.05 *versus* AAC+PYR.

### Pyridostigmine treatment inhibited cardiac hypertrophy

**Figure 4:**
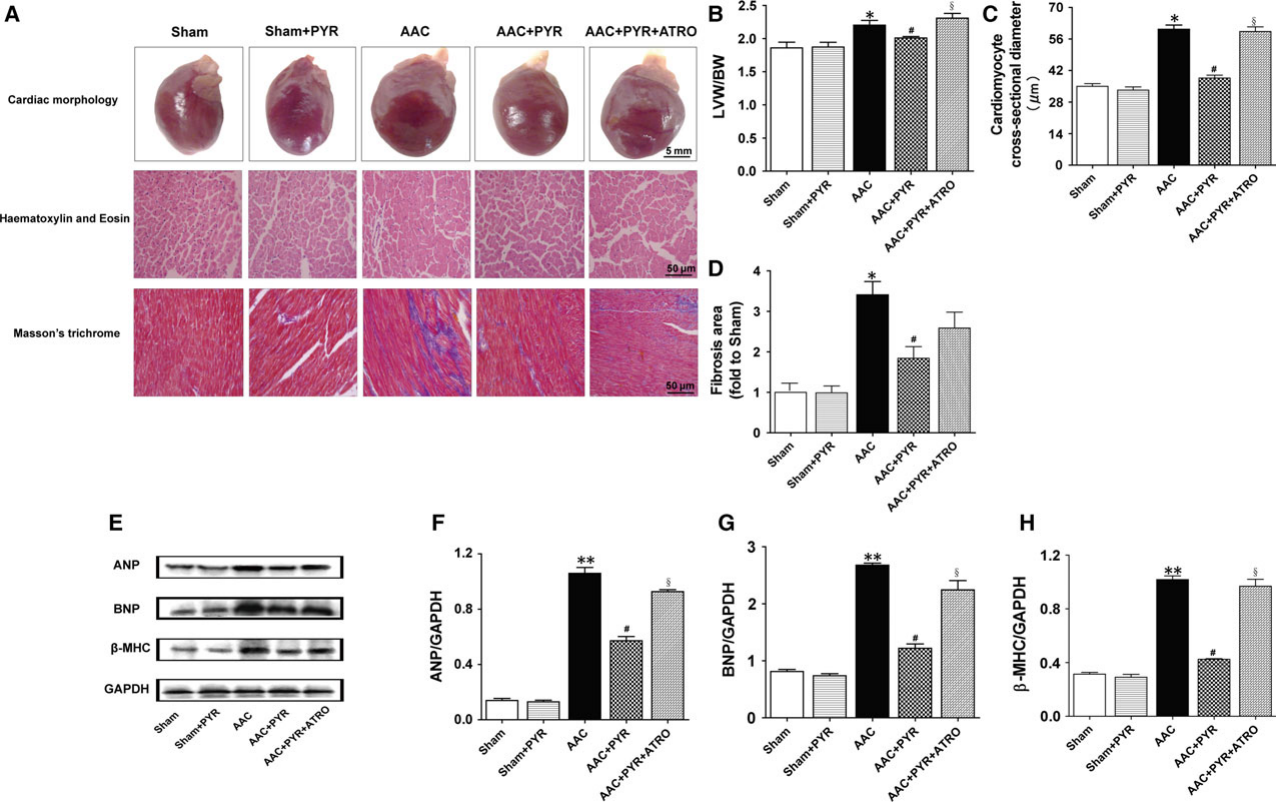
Pyridostigmine reduced cardiac hypertrophy in rats with pressure overload. (**A**) Representative changes in cardiac morphology, cardiomyocyte enlargement and collagen deposition. Scale bars are 5 mm, 50 μm and 50 μm. (**B**) Left ventricular weight‐to‐body weight ratio. (**C**) Cross section of cardiac myocytes in the left ventricle. (**D**) Area of cardiac fibrosis as determined by Masson's trichrome staining. (**E**) Expression levels of markers of cardiac hypertrophy. (**F–H**) ANP, BNP and β‐MHC protein levels. Data are means ± S.E.M. (*n* = 7). *P < 0.05, **P < 0.01 *versus* sham. #*P* < 0.05 *versus* AAC, §*P* < 0.05 *versus* AAC+PYR.

The effects of pyridostigmine on cardiac hypertrophy are shown in Table 1 and Figure 4. LVED(S)V, IVSd(s), LVIDd(s), LVPWd(s), LVW/BW and the myocardial cell diameter markedly increased in rats with pressure overload; these increases were reversed by treatment with pyridostigmine. The cardiac fibrosis area differed markedly between the sham and AAC groups (Fig. 4A and D). Eight weeks of treatment with pyridostigmine reversed these pathological changes. Cardiac function and fibrosis of the left ventricle was almost identical in the sham and sham+PYR groups. Atropine abolished the effect of pyridostigmine on cardiac function in the AAC group,but it had no effect on cardiac function and fibrosis in sham rats treated with pyridostigmine (Table 1, Table S1, Fig. S2 and Fig. S3).

Constriction of the abdominal aorta also increased the levels of cardiac hypertrophy‐related molecular markers (Fig. 4E). The protein levels of ANP, BNP and β‐MHC in LV tissue were higher in the AAC than sham groups. These increases were prevented by pyridostigmine; however, the protective effects of pyridostigmine were partly blocked by atropine. There was no significant difference in ANP, BNP or β‐MHC expression between the sham and sham+PYR groups.

### Pyridostigmine administration inhibited the CaN/NFAT3/GATA4 signalling pathway

**Figure 5:**
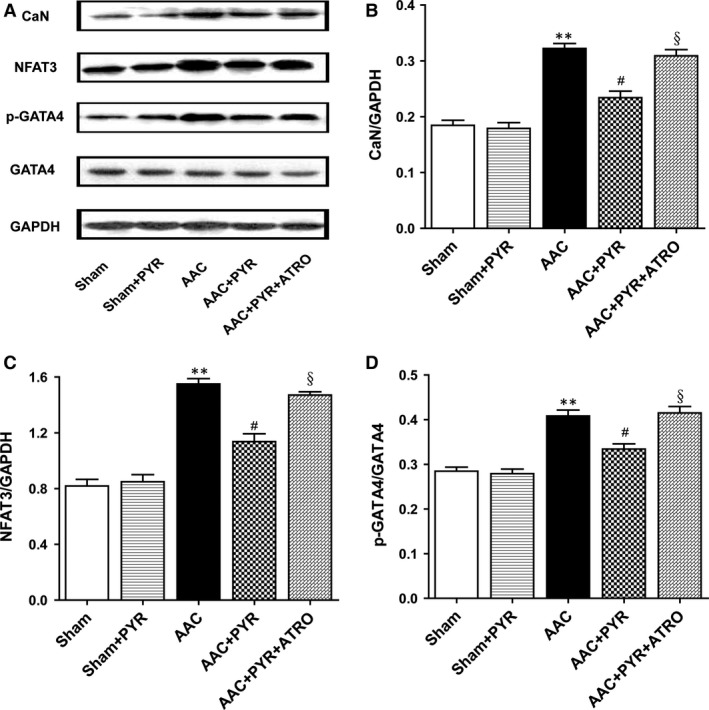
Pyridostigmine inhibited the CaN/NFAT3/GATA4 signalling pathway. (**A**) Representative Western blots for CaN, NFAT3, GATA4 and p‐GATA4. (**B–D**) Western blot analysis of CaN, NFAT3 and p‐GATA4 expression. Data are means ± S.E.M. (*n* = 7). **P < 0.01 *versus* sham. #*P* < 0.05 *versus* AAC, §*P* < 0.05 *versus* AAC+PYR.

Western blot analysis was performed to assess the role of CaN/NFAT3/GATA4 in cardiac hypertrophy. The expression of CaN, NFAT3 and p‐GATA4, but not of total GATA4, was significantly higher in the AAC than sham group (Fig. 5). Pyridostigmine decreased CaN, NFAT3 and p‐GATA4 expression in the AAC+PYR group compared with the AAC group. No marked increases in CaN, NFAT3 or p‐GATA4 protein levels were observed in the sham+PYR *versus* sham group. Atropine abolished the effect of pyridostigmine on CaN/NFAT3/GATA4 in cardiac hypertrophy.

### Pyridostigmine inhibited the interaction between Orai1 and STIM1

**Figure 6:**
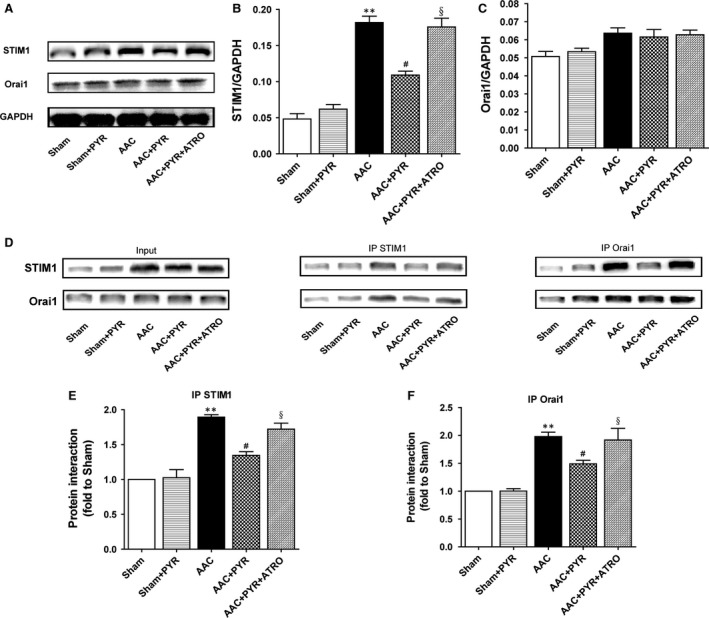
Treatment with pyridostigmine inhibited Orai1/STIM1 complex formation. (**A–C**) Orai1 and STIM1 protein levels in cardiac tissue. (**D–F**) Immunoprecipitation assay of the interaction between Orai1 and STIM1. Data are means ± S.E.M. (*n* = 7). **P < 0.01 *versus* sham, #*P* < 0.05 *versus* AAC, §*P* < 0.05 *versus* AAC+PYR.

The expression of STIM1, but not Orai1, increased significantly in the AAC group (Fig. 6B and C). Co‐immunoprecipitation assays showed that the amount of Orai1/STIM1 complex was increased in rats with pressure overload (Fig. 6E and F). Treatment with pyridostigmine inhibited the interaction between Orai1 and STIM1, and this effect was blocked by atropine.

## Discussion

**Figure 7:**
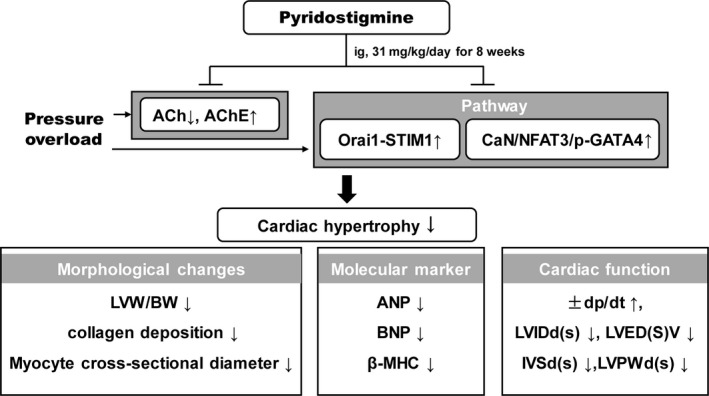
Treatment with pyridostigmine attenuated cardiac hypertrophy. In the present study, we found that vagal discharge was significantly lower in cardiac hypertrophy induced by pressure overload. Pyridostigmine attenuated cardiac hypertrophy and improved cardiac function *via* a mechanism involving improvement of cholinergic transmission efficiency and inhibition of the Orai1/STIM1/CaN/NFAT3/p‐GATA4 signalling pathway. ACh, acetylcholine; AChE, acetylcholinesterase; LVW/BW, left ventricular weight/body weight; ANP, atrial natriuretic peptide; BNP, brain natriuretic peptide; β‐MHC, β‐myosin heavy chain; ±dp/dt, maximum slope of systolic pressure increment and diastolic pressure decrement; LVIDd(s), left ventricular internal dimension in systole and diastole; LVED(S)V, end‐systolic and end‐diastolic left ventricular volumes; IVSd(s), thickness of the interventricular septum; LVPWd(s), thickness of the left ventricular posterior wall in systole and diastole.

In this study, we analysed cardiac function and vagal activity to evaluate the effect of long‐term pyridostigmine treatment in rats with pressure overload. The major findings include the following: (*i*) pressure overload led to cardiac hypertrophy, LV dysfunction and hemodynamic impairment, with a significant reduction in vagal discharge; (*ii*) treatment with pyridostigmine improved the cholinergic transmission efficiency possibly *via* inhibiting the activity of AChE and increasing the concentration of ACh, without affecting vagal discharge, and prevented cardiac hypertrophy as evidenced by the reduced LV weight/body weight ratio, collagen deposition, ventricular thickness and expression of biomarkers of cardiac hypertrophy; and (*iii*) pyridostigmine inhibited the CaN/NFAT3/GATA4 signalling pathway and suppressed Orai1/STIM1 complex formation (Fig. 7). Thus, our findings indicate that pyridostigmine may be an important therapeutic strategy for heart failure.

Data obtained from previous studies suggest that cardiac autonomic dysfunction, which is characterized by enhanced sympathetic activity and withdrawal of parasympathetic control, occurs in several cardiovascular diseases 21, 22, 23. Yusuf *et al*. showed that β‐blockers can prevent sympathetic hyperactivity and decrease cardiovascular mortality in patients with heart failure 24. Therapeutic options to improve vagal activity have been a focus of recent studies 25, 26. These therapeutic strategies include direct vagal activation, pharmacological modulation and exercise training 4. Pyridostigmine, a reversible anticholinesterase agent, does not cross the blood–brain barrier and acts in the peripheral synaptic cleft, which could increase HR recovery after exercise and improve haemodynamic profiles during dynamic exercise in patients with heart failure 27, 28. These results indicate that pyridostigmine might protect against cardiovascular diseases by improving the vagal activity. Meanwhile, our study showed that treatment with pyridostigmine or pyridostigmine plus atropine had no significant effects on cardiac function and cardiac morphology compared with the sham group. Similarly, Potes *et al*. and Eger *et al*. have shown that pyridostigmine or atropine itself has no significant effect on cardiac function in man 29, 30. Thus, pyridostigmine administration, and the subsequent inhibition of ACh, may be a promising therapeutic strategy for vagal modulation in patients with cardiovascular diseases without direct affecting cardiac function in healthy individuals. Recent studies have also shown that pyridostigmine decreases sympathetic drive in models of cardiovascular diseases 31, 32. Manabe *et al*. showed that ACh released from vagal ending has suppressive effects on norepinephrine released from cardiac sympathetic nerves 33, 34. Meanwhile, ACh affects muscarinic receptors located pre‐synaptically in sympathetic nerve endings, thereby inducing a decrease in norepinephrine release 35, 36. In the present study, pyridostigmine can increase ACh levels by inhibiting AChE activity. Thus, pyridostigmine may suppress the sympathetic nervous system by influencing sympathetic signalling and muscarinic receptors located in the cardiac tissue.

Our present experiments provide the first evidence to indicate that pyridostigmine can increase the levels of ACh without affecting vagal discharge. It is possible that pyridostigmine has this effect because it is a reversible AChE inhibitor; by inhibiting the activity of AChE, pyridostigmine increases ACh concentration. In addition, pyridostigmine, which has a quaternary carbamine group, does not cross the blood–brain barrier, and cannot have any significant central influence on vagal discharge 37. Pyridostigmine, therefore, must primarily act in the peripheral synaptic cleft to inhibit the hydrolysis of ACh released by cholinergic neurons.

Previous studies have shown that cardiomyocyte‐secreted ACh plays multiple roles in ventricular performance 38, 39. Roy *et al*. has demonstrated that the long‐term exposure of cardiomyocytes to lower levels of ACh induces a hypertrophic response 40. Our study showed that pyridostigmine could enhance serum levels of ACh and attenuate cardiomyocyte hypertrophy. In addition, another recent study showed that there was significant induction of vesicular acetylcholine transporters in LV myocytes in a model of sympathetic hyperactivity. Treatment with pyridostigmine restored ACh availability and lowed vesicular acetylcholine transporter levels, which had a beneficial effect on cardiac remodelling 41. In a recent study, they also showed that genetic mutations in machinery regulating the synthesis and transportation of ACh are deleterious for cardiac remodelling induced by chronic exposure to angiotensin II 42. Generally, the factors affecting ACh levels include the synthesis, transportation and hydrolysis of ACh. In this study, we focused on the hydrolysis of ACh. Our results showed that pyridostigmine could increase ACh levels by inhibiting ACh hydrolysis, and could ameliorate cardiac hypertrophy in rats with pressure overload. Roy's and our results confirmed similar evidence that improved cardiac cholinergic transmission helps to inhibit the ventricular remodelling and dysfunction *in vivo* from different angles.

We also found that treatment with pyridostigmine reduced LVSP and LVEDP but increased ±dp/dt in the AAC group. The improvement of cardiac function by pyridostigmine administration may be mediated by a reduction in ventricular stress, in agreement with a previous report 10. Interestingly, there was no significant difference in HR among groups in the present study. Some studies have revealed a pyridostigmine‐induced increase in HR after the removal of adrenergic and cholinergic influences on the heart by propranolol and methylatropine in models of sympathetic hyperactivity‐induced cardiac dysfunction 43, 44. These data indicate that pyridostigmine itself may increase HR, which may counteract the reduction of HR induced by ACh. The effect of pyridostigmine on HR may be not related to the level of ACh. Previous studies have not found significant differences in HR between groups; our findings are in line with those results 32, 41.

Cardiac hypertrophy induced by pressure overload is defined as an increase in ventricular myocardial mass, ventricular wall thickness and ventricular volume. Myocardial size was a very important maker to evaluate cardiac hypertrophy in several studies 45, 46. In the present study, all rats were killed under anaesthesia induced by intraperitoneal injection of pentobarbital sodium. After that, each rat heart was harvested and prepared for histologic examination. However, there was some limitation in the current study. In our method, cardiomyocytes might not be at the same stage of cardiac cycle. In fact, the killing of the animals with injection of KCl solution allows the evaluation of cardiomyocyte size at the same phase of cardiac cycle 47. In this study, pyridostigmine significantly inhibited pathological ventricular wall thickness and ventricular volume. In addition, pyridostigmine reduced the changes in cardiac morphology, myocardial cell diameter and collagen deposition, in agreement with previous findings based on other models of sympathetic hyperactivity‐induced cardiac dysfunction 41. Moreover, pyridostigmine decreased the expression of ANP, BNP and β‐MHC in cardiomyocytes, which further suggests an inhibitory effect of pyridostigmine on cardiac hypertrophy.

Activation of CaN plays a crucial role in pathological cardiac hypertrophy 48. CaN dephosphorylates NFAT3, which translocates to the nucleus from the cytosol, where it interacts with GATA4 to initiate hypertrophic gene transcription 14. Ca^2+^ channel dysfunction may also be a factor in cardiac hypertrophy induced by pressure overload 49, 50. STIM1, which acts as a Ca^2+^ sensor in the lumen of the endoplasmic reticulum/sarcoplasmic reticulum, aggregates into oligomers and clusters proximal to the plasma membrane to interact with Orai to facilitate Ca^2+^ influx 51. Luo *et al*. also showed that STIM1 is a key regulator of the CaN–NFAT signalling pathway during the response to hypertrophic agonists 52. In our study, pyridostigmine inhibited the expression of CaN, NFAT3, p‐GATA4 and STIM1, prevented Orai1/STIM1 complex formation and attenuated cardiac hypertrophy. Therefore, pyridostigmine may inhibit the activation of CaN/NFAT3 through the downregulation of STIM1. Interestingly, there was no significant difference in Orai1 expression among the groups, but a greater quantity of Orai1 co‐precipitated with STIM1 in the AAC group. In contrast, Youakim *et al*. reported that Orai3 but not Orai1 is an essential partner of STIM1 for the promotion of cardiac hypertrophy in cardiomyocytes 53. This is likely because Orai1, the major Orai family subtype, has a stronger binding affinity for STIM1 than Orai3.

A recent study demonstrated that Orai1/STIM1 may be upstream of the CaN/NFAT3/GATA4 signalling pathway, which is involved in the progress of cardiac hypertrophy 16. Orai1 overexpression or STIM1 overexpression results in cardiac hypertrophy through activation of the CaN/NFAT signalling pathway 18, 54. In contrast, the deletion of Orai1 or STIM provides cardioprotection in rats with pressure overload by inhibiting the CaN/NFAT signalling cascade 18, 52. In the present study, pyridostigmine may have suppressed the formation of Orai1/STIM1 complex, resulting in inhibition of the activation of the CaN/NFAT3/GATA4 signalling pathway and attenuation of cardiac hypertrophy. The inhibitory effect of pyridostigmine on the CaN/NFAT3/GATA4 signalling pathway plays important roles in cardiac hypertrophy. Previous studies have shown that the activation of the renin angiotensin system is one of the triggers for the activation of the CaN/NFAT3/GATA4 pathway 18, 54. Our recent study demonstrated that administration of pyridostigmine decreases the levels of angiotensin II and angiotensin II type 1 receptor in cardiac tissue 55. Therefore, pyridostigmine may inhibit the CaN/NFAT3/GATA4 signalling pathway by suppressing the activation of the renin angiotensin system, resulting in amelioration of cardiac hypertrophy. Meanwhile, activation of the sympathetic nerve also plays a critical role in the activation of the CaN/NFAT3/GATA4 signalling pathway. Pyridostigmine reduces sympathetic drive in models of cardiovascular diseases. The effect of pyridostigmine on the sympathetic nerve may lead to CaN/NFAT3/GATA4 signalling cascade inhibition and subsequent amelioration of cardiac hypertrophy. Our data suggest that cholinergic stimulation with pyridostigmine may play a critical role in the prevention of cardiac hypertrophy.

In conclusion, we found that vagal discharge was significantly reduced in cardiac hypertrophy induced by pressure overload. Treatment with pyridostigmine attenuated cardiac hypertrophy and improved cardiac function, which was related to improvement of cholinergic transmission efficiency *via* inhibition of AChE, suppression of Orai1/STIM1 complex formation and inhibition of the downstream CaN/NFAT3/p‐GATA4 signalling pathway in the pathogenesis of cardiac conditions in the presence of stress.

## Conflict of interest

The authors declared no conflict of interest.

## Supporting information


**Table S1** Echocardiographic assessment of cardiac function after treatment with pyridostigmine or pyridostigmine plus atropine in rats.
**Figure S1** Effect of pyridostigmine or pyridostigmine plus atropine on serum AChE activity in rats.
**Figure S2** Representative pictures of echocardiography in the sham, sham+PYR and sham+PYR+ATRO groups.
**Figure S3** Effect of pyridostigmine or pyridostigmine plus atropine on cardiac morphology and histology in rats.
