# Supplementary material for: Long‐term administration of pyridostigmine attenuates pressure overload‐induced cardiac hypertrophy by inhibiting calcineurin signalling
Source: J Cell Mol Med. 2017 Mar 10;21(9):2106–16. doi: 10.1111/jcmm.13133 (PMC5571547; doi:10.1111/jcmm.13133)
Supplement: Supplementary file 1 — Table S1 Echocardiographic assessment of cardiac function after treatment with pyridostigmine or pyridostigmine plus atropine in rats. Figure S1 Effect of pyridostigmine or pyridostigmine plus atropine on serum AChE activity in rats. Figure S2 Representative pictures of echocardiography in the sham, sham+PYR and sham+PYR+ATRO groups. Figure S3 Effect of pyridostigmine or pyridostigmine plus atropine on cardiac morphology and histology in rats. [file JCMM-21-2106-s001.docx]

**Long-term administration of pyridostigmine attenuates pressure overload-induced cardiac hypertrophy by inhibiting calcineurin signaling**

Yi Lu, Ming Zhao, Jin-Jun Liu, Xi He, Xiao-Jiang Yu, Long-Zhu Liu, Lei Sun, Li-Na Chen*, Wei-Jin Zang*

Department of Pharmacology School of Basic Medical Sciences Xian Jiaotong University Health Science Center, Xi’an, Shaanxi, 710061, People Republic of China

***Corresponding Author**: Li-Na Chen, Ph.D. and Wei-Jin Zang, Ph.D.

Address: P.O. Box 77^#^, No.76 Yanta West Road, Department of Pharmacology, Xi’an Jiaotong University Health Science Center, Xi'an, People Republic of China, 710061.

Tel: +86-29-82655150

E-mail: [chenlin@xjtu.edu.cn](mailto:chenlin@xjtu.edu.cn) and [zwj@xjtu.edu.cn](mailto:zwj@xjtu.edu.cn)

**Running title**: Pyridostigmine ameliorates cardiac hypertrophy

**Supplementary Table 1. Echocardiographic assessment of cardiac function after treatment with pyridostigmine or pyridostigmine plus atropine in rats.**

|  | **Sham** | **Sham+PYR** | **Sham+PYR+ATRO** |
| --- | --- | --- | --- |
| EF(%) | 71.39±3.85 | 67.97±2.52 | 69.06±2.65 |
| FS(%) | 43.39±3.88 | 39.48±2.13 | 40.30±2.11 |
| HR(bpm) | 353.16±7.66 | 331.23±8.87 | 352.61±8.44 |
| SVR(dyne*sec*cm^-5^) | 136.65±5.26 | 156.74±11.46 | 152.98±13.10 |
| LVIDd(mm) | 7.29±0.17 | 7.21±0.18 | 7.05±0.10 |
| LVIDs(mm) | 4.17±0.33 | 4.39±0.18 | 4.21±0.18 |
| LVDV(μl) | 281.16±13.54 | 278.25±13.25 | 260.17±7.38 |
| LVSV(μl) | 83.91±13.08 | 89.51±8.91 | 81.22±8.02 |
| LVPWd(mm) | 1.69±0.12 | 1.65±0.07 | 1.51±0.09 |
| LVPWs(mm) | 2.75±0.18 | 2.50±0.12 | 2.40±0.17 |
| IVSd(mm) | 1.66±0.10 | 1.73±0.14 | 1.85±0.08 |
| IVSs(mm) | 2.79±0.10 | 2.69±0.13 | 2.73±0.08 |

Treatment with pyridostigmine or pyridostigmine plus atropine for 6 weeks did not affect cardiac function in rats. EF, left ventricular ejection fraction; FS, left ventricular fractional shortening; HR, heart rate; SVR, systemic vascular resistance; LVIDd(s), left ventricular internal dimension in systole and diastole; LVD(S)V, left ventricle volume diastole (systole); IVSd(s), thickness of the interventricular septum; LVPWd(s), thickness of the left ventricular posterior wall in systole and diastole. Data are shown as the means ± standard error of the mean (n = 10).

**
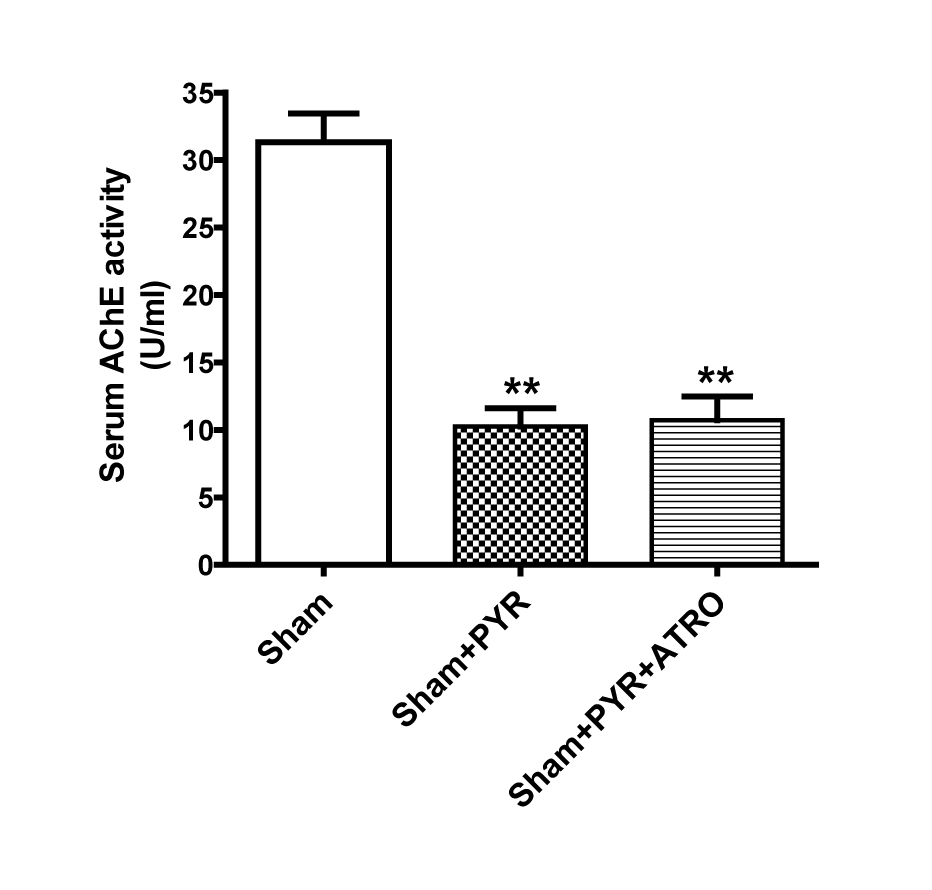
**

**Figure S1. Effect of pyridostigmine or pyridostigmine plus atropine on serum AChE activity in rats.** Serum AChE activity was reduced after 6 weeks of treatment with pyridostigmine or pyridostigmine plus atropine in rats. Data are means ± standard errors of the mean (n = 10). ^**^*p*<0.01*vs.* sham.


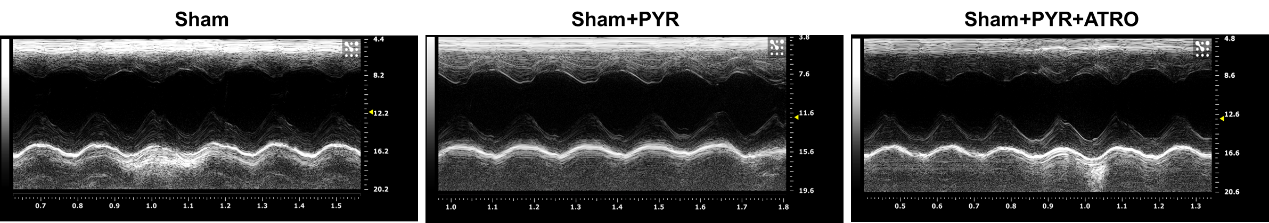


**Figure S2. Representative pictures of** **enchocardiography in the sham, sham+PYR and sham+PYR+ATRO groups.**

**
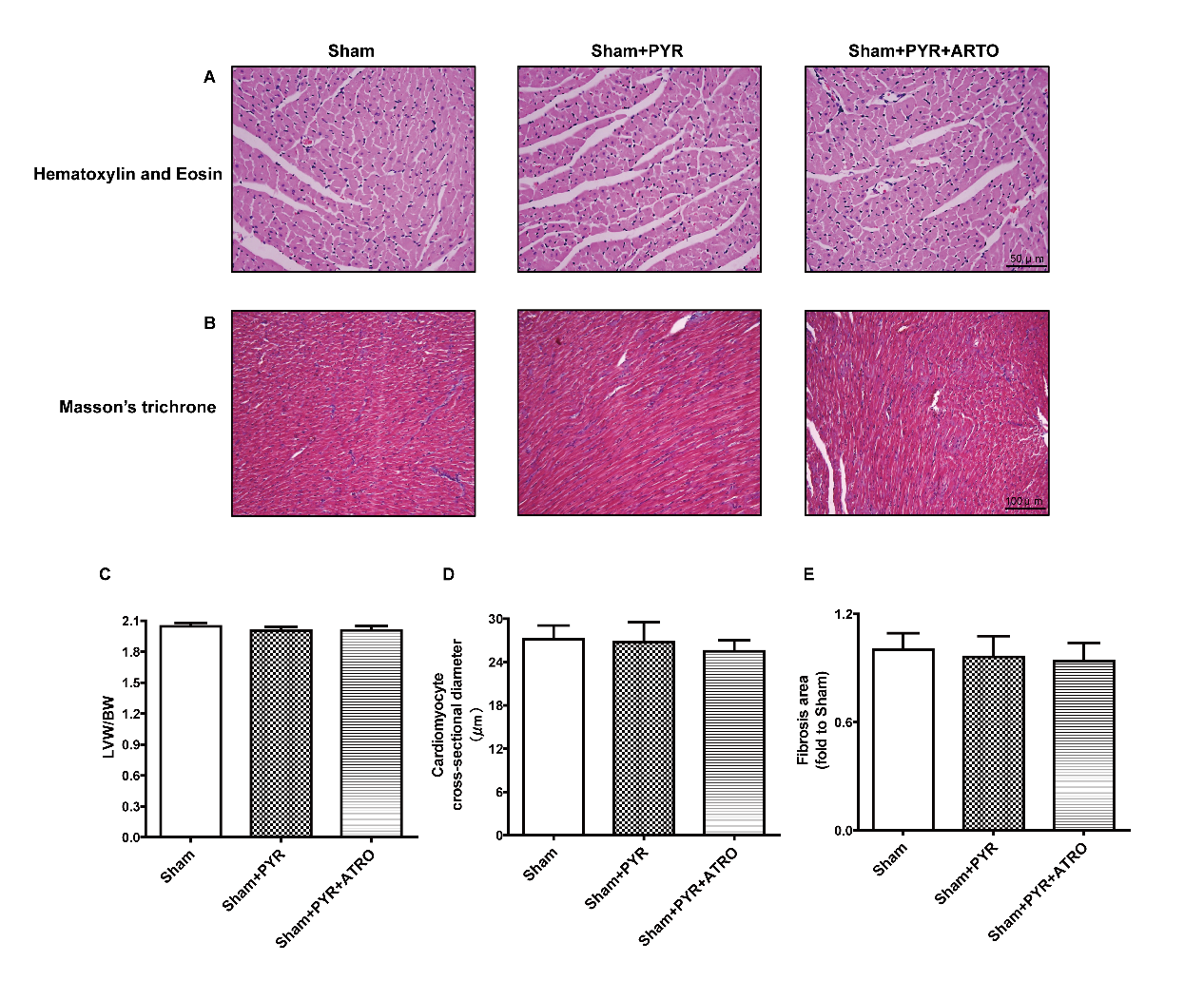
**

**Figure S3. Effect of pyridostigmine or pyridostigmine plus atropine on cardiac morphology and histology in rats.** No significant differences was observed among the groups after 6 weeks of treatment with pyridostigmine or pyridostigmine plus atropine. (A, B) Representative changes in cardiomyocyte enlargement and collagen deposition. Scale bars are 50 μm and 100 μm, respectively. (C) Left ventricular weight to body weight ratio. (D) Cross-section of cardiac myocytes in the left ventricle. (E) Area of cardiac fibrosis as determined by Masson’s trichrome staining. Data are means ± standard errors of the mean (n = 10).
